# Audio-visual onset differences are used to determine syllable identity for ambiguous audio-visual stimulus pairs

**DOI:** 10.3389/fpsyg.2013.00331

**Published:** 2013-06-26

**Authors:** Sanne ten Oever, Alexander T. Sack, Katherine L. Wheat, Nina Bien, Nienke van Atteveldt

**Affiliations:** ^1^Faculty of Psychology and Neuroscience, Maastricht UniversityMaastricht, Netherlands; ^2^EMACS Research Unit, University of LuxembourgLuxembourg, Luxembourg; ^3^Neuroimaging and Neuromodeling Group, Netherlands Institute for NeuroscienceAmsterdam, Netherlands

**Keywords:** audiovisual, temporal cues, audio-visual onset differences, content cues, predictability, detection

## Abstract

Content and temporal cues have been shown to interact during audio-visual (AV) speech identification. Typically, the most reliable unimodal cue is used more strongly to identify specific speech features; however, visual cues are only used if the AV stimuli are presented within a certain temporal window of integration (TWI). This suggests that temporal cues denote whether unimodal stimuli belong together, that is, whether they should be integrated. It is not known whether temporal cues also provide information about the identity of a syllable. Since spoken syllables have naturally varying AV onset asynchronies, we hypothesize that for suboptimal AV cues presented within the TWI, information about the natural AV onset differences can aid in speech identification. To test this, we presented low-intensity auditory syllables concurrently with visual speech signals, and varied the stimulus onset asynchronies (SOA) of the AV pair, while participants were instructed to identify the auditory syllables. We revealed that specific speech features (e.g., voicing) were identified by relying primarily on one modality (e.g., auditory). Additionally, we showed a wide window in which visual information influenced auditory perception, that seemed even wider for congruent stimulus pairs. Finally, we found a specific response pattern across the SOA range for syllables that were not reliably identified by the unimodal cues, which we explained as the result of the use of natural onset differences between AV speech signals. This indicates that temporal cues not only provide information about the temporal integration of AV stimuli, but additionally convey information about the identity of AV pairs. These results provide a detailed behavioral basis for further neuro-imaging and stimulation studies to unravel the neurofunctional mechanisms of the audio-visual-temporal interplay within speech perception.

## Introduction

Although audition is our main informant during speech perception, visual cues have been shown to strongly influence identification and recognition of speech (Campbell, 2008). Visual cues are used to increase understanding, especially in noisy situations when auditory information alone is not sufficient (Sumby and Pollack, 1954; Bernstein et al., 2004; Grant et al., 2004). It is known that temporal, spatial, and semantic cues in visual signals are used to improve auditory speech perception (Wallace et al., 1996; Stevenson and James, 2009). However, it is largely unknown how these different cues are combined to create our auditory percept. In the current research, we used semantically congruent or incongruent audio-visual syllables presented with varied stimulus onset asynchronies (SOAs) between the auditory and visual stimuli, to investigate the interaction between temporal and content factors during audio-visual speech perception (see e.g., Vatakis and Spence, 2006; van Wassenhove et al., 2007; Vatakis et al., 2012). Specifically, we were interested whether natural onset asynchronies inherent to audio-visual syllable pairs influence syllable identification.

Often, stop-consonant syllables (e.g., /ba/ and /da/) are used to examine syllable identification (see e.g., McGurk and MacDonald, 1976; van Wassenhove et al., 2007; Arnal et al., 2011). Stop consonants are consistent in the manner in which they are produced (the vocal tract is blocked to cease airflow), but vary in the type and amount of identity information conveyed by the visual and auditory channels. Specifically, whether or not the vocal tract is used to produce a consonant (i.e., the voicing of a sound, /ba/ vs. /pa/) is not visible, since the vocal tract is located in the throat. Therefore, the auditory signal is more reliable than the visual signal in determining the voicing of a speech signal (Wiener and Miller, 1946; McGurk and MacDonald, 1976). On the other hand, which part of the mouth we use for producing a syllable is mostly a visual signal. For example, uttering a syllable with our lips (like /ba/) vs. our tongue (like /da/) is more visible than audible. Visual speech thus conveys mostly information about the place of articulation (POA) of the sound, and adding acoustic noise to a spoken syllable makes the POA particularly difficult to extract on basis of auditory information (Wiener and Miller, 1946; McGurk and MacDonald, 1976; van Wassenhove et al., 2005). However, the amount of visual information about the POA varies for different syllables: bilabial syllables (pronounced with the lips) are better dissociated than coronal and dorsal syllables (pronounced with the front or body of the tongue, respectively). Thus, it seems that auditory and visual speech signals are complementary in identifying a syllable, since voicing information is best conveyed by auditory cues and POA information by visual cues (Summerfield, 1987; Campbell, 2008).

Auditory and visual stimuli can be linked based on their content information; the information about the identity (the “what”) of a stimulus. We will continue to use the term content information, although in other studies the term semantic information is also used (for a review, see Doehrmann and Naumer, 2008). The amount of content information conveyed by a unimodal signal is variable, for different stimuli (as explained above) as well as for individuals perceiving the same stimuli, and the reliability of the information determines how strongly it influences our percept (Driver, 1996; Beauchamp et al., 2004; van Wassenhove et al., 2005; Blau et al., 2008). For example, the amount of content information present in visual speech signals is widely variable, as reflected in individual differences in lipreading skills (MacLeod and Summerfield, 1987; Auer and Bernstein, 1997), and it has been shown that more profound lipreaders also use this information more (Pandey et al., 1986; Auer and Bernstein, 2007). Additionally, visual speech signals that convey more content information (like bilabial vs. dorsal syllables, as explained above) bias the speech percept more strongly (McGurk and MacDonald, 1976; van Wassenhove et al., 2005). However, the influence of visual information on auditory perception often depends not only on the nature and quality of the visual signal, but also on the quality of the auditory signal, since visual input is especially useful for sound identification when background noise levels are high (Sumby and Pollack, 1954; Grant et al., 2004). Thus, during audiovisual identification unimodal cues seem to be weighted based on their reliability, to create the audio-visual percept (Massaro, 1987, 1997). Additionally, the amount of weight allocated to each modality depends not only on the overall quality of the signal, but also on the reliability of the signal for the specific feature that needs to be identified. For example, spatial perception is more accurate in the visual domain, therefore spatial localization of audio-visual stimuli mostly dependents on visual signals (Driver, 1996). One of the aims of our study was to provide further support for the notion that reliable modalities are weighted more heavily (Massaro, 1997; Beauchamp et al., 2004). Specifically, we investigated whether systematic difference in the reliability of the voicing and POA features of the syllable (see above) biases which modality is weighted more heavily.

The main aim of our study was to investigate how the temporal relation between audio-visual pairs influences our percept. It is known that auditory and visual signals are only integrated when they are presented within a certain temporal window (Welch and Warren, 1986; Massaro et al., 1996; Ernst and Bülthoff, 2004), this is the so-called temporal window of integration (TWI). The TWI is for example measurable with synchrony judgments, in which temporal synchrony of audio-visual signals is only perceived if audio-visual pairs are presented within a certain range of onset asynchronies (Meredith et al., 1987; Spence and Squire, 2003). The TWI highlights that the temporal relationship of auditory and visual inputs is another important determinant for integration, in addition to information about the “what” of a stimulus. The importance of this window has been replicated many times for perceptual as well as neuronal integration (Stein and Meredith, 1993; van Atteveldt et al., 2007; van Wassenhove et al., 2007). Typical for the TWI is that the point of maximal integration occurs with visual stimuli leading (Zampini et al., 2003). This seems to relate to the temporal information visual signals provide, namely a prediction of the “when” of the auditory signal, since they naturally precede the sounds (Chandrasekaran et al., 2009; Zion Golumbic et al., 2013). However, the difference between the onset of the visual and auditory signal varies across syllables (Chandrasekaran et al., 2009) and it is not known whether these natural onset differences can cue the identity of the speech sound. It has been shown that monkey auditory cortex and superior temporal cortex are sensitive to natural audio-visual onset differences in monkey vocals (Ghazanfar et al., 2005; Chandrasekaran and Ghazanfar, 2009). In humans, it has been shown that onset differences within the auditory modality are used to identify auditory syllables (Miller, 1977; Munhall and Vatikiotis-Bateson, 1998). For example, the distinction between a voiced or unvoiced syllable in the auditory signal is solely based on onset differences of specific frequency bands. However, it is not known whether audio-visual onset information is used to identify speech sounds. We hypothesize that inherent onset differences between auditory and visual articulatory cues can be used to identify spoken syllables. Specifically, we hypothesize that coronal (e.g., /da/) and dorsal (e.g., /ga/) stimuli (pronounced with the front or body of the tongue, respectively) might have audio-visual onset difference, in which dorsal stimuli produce longer onset differences due to a longer distance from the POA to the external, audible sound.

Traditionally, only a single dimension in the auditory or visual signal is altered to investigate the influence of visual cues. However, more and more studies are showing interactions between different crossmodal cues. For example, Vatakis and Spence (2007) found that if the gender of a speaker is incongruent for auditory and visual speech, less temporal discrepancy is allowed for the stimuli to be perceived as synchronous. Stimuli in the McGurk effect (McGurk and MacDonald, 1976), in which an auditory [ba], presented with an incongruent visual /ga/ is perceived as a /da/, are also perceived as synchronous for a narrower temporal window, compared to congruent audio-visual syllables (van Wassenhove et al., 2007). Furthermore, in recent work we showed that auditory detection thresholds are lower if temporal predictive cues are available in both the auditory and visual domain (ten Oever et al., submitted). In addition, interactions between semantic relatedness and spatial processing have been reported (Driver, 1996; Parise and Spence, 2009; Bien et al., 2012), as well as interactions between temporal and spatial factors (Stevenson et al., 2012). However, it is still unknown how interactions between auditory and visual content as well as temporal cues influence speech identification.

In sum, for stop consonants, auditory cues provide content information with regard to voicing, whereas visual cues provide content information with regard to POA (with varying reliability, e.g., for bilabial vs. dorsal/coronal). Therefore, we were able to make use of these properties in order to investigate whether incongruent pairs of stimuli are identified depending on the modality that has the most reliable information for the specific features; POA and voicing. Additionally, we used different SOAs to investigate the temporal profile of this effect. Specifically, we were interested in the temporal window in which visual information influences the auditory percept, and whether ambiguity in the identity of auditory syllables can be resolved using differences in natural audio-visual onsets in speech.

## Materials and methods

### Participants

Eight healthy native Dutch volunteers (3 male, mean age 20.9, SD 2.6) participated in the study. All participants reported to have normal hearing and normal or corrected to normal vision. Participants were unaware of the goal of the study before they completed the experiment. Informed consent was given before participating. Ethical approval was given by the Ethical Committee of the Faculty of Psychology at the University of Maastricht. Participants received €40 or student participation credits in compensation for their time.

### Stimulus material

Six Dutch syllables, pronounced by a native Dutch female speaker, were used as auditory and visual stimuli (/pa/, /ba/, /ta/, /da/, /ka/, /ga/). For variability, we recorded three different versions of every syllable. Sounds were digitized at 44.1 kHz, with 16-bit amplitude resolution and were equalized for maximal intensity. Videos had a digitization rate of 30 frames per second and were 300 × 300 pixels. We used a method similar to method used in van Wassenhove et al. (2005) to create the videos. Videos lasted 2367 ms, including a fade in of a still face (8 frames), the still face (5 frames), the mouth movements (52 frames), and a fade out of a still face (5 frames). MATLAB (Mathworks) scripts were used to create these videos. Additionally, for every stimulus there was a still face video with the fade out and fade-in frames. First, we tested three participants with SOAs between auditory and visual stimuli ranging from VA (visual lead) 300 ms up to AV (auditory lead) 300 in steps of 30 ms, since this range covers the TWI for syllables used before (see e.g., van Wassenhove et al., 2007; Vatakis and Spence, 2007). However, for the extreme VA and AV SOAs participants still seemed to use the visual information to determine their responses, therefore we chose to widen the SOA range (ranging from VA 540 to AV 540 ms in steps of 60 ms for the other participants). To align the incon-gruent auditory stimuli with the videos, the maximal intensity of the incongruent auditory stimulus was aligned with the congruent auditory stimulus.

### Procedure

Each participant was tested in two separate experimental sessions, both lasting 2 h. In the first session a staircase, a unimodal visual experiment, and the first part of the audio-visual experiment was conducted. The second session consisted of the remainder of the audio-visual experiment.

The staircase procedure consisted of a six-alternatives forced choice procedure in which participants were asked to identify the six different syllables without presentation of the videos. Syllables were randomly presented over a background of white noise. Depending on the accuracy of the response, the intensity of the white noise was increased or decreased for the next trial. A two-up, one-down procedure (Levitt, 1971) with a total of 20 reversals was employed, which equals approximately 70% identification threshold. The individually obtained white noise intensity was used in the following experiments as background noise for the individual participants.

In the unimodal visual experiment participants were requested to recognize the identity of the syllable based on the videos only. White noise was presented as background noise. First, a fixation cross was presented for 800 ms, followed by a syllable video. Finally, a question mark was presented with the six possible response options to which participants were requested to respond. After participants responded there was a 200-ms break before the next trial started. In total, 360 stimuli were presented, 60 per syllable in 4 separate blocks.

The audio-visual experiment had a similar trial configuration to the unimodal visual experiment, but consisted of the presentation of audio-visual pairs. Only two visual stimuli were used here; /pa/ and /ga/. These specific syllables were selected because they differ from each other in terms of POA: /pa/ is a bilabial syllable, pronounced in the front of the mouth, whereas /ga/ is dorsal syllable, pronounced in the back of the mouth. Furthermore, it has been shown that identifying /pa/ is much easier than /ga/ (Wiener and Miller, 1946; McGurk and MacDonald, 1976; van Wassenhove et al., 2005), thus serving our aim to manipulate the amount of information provided by the visual stimulus. Participants were instructed to identify the auditory stimulus only (again choosing between the six possible response options), while ignoring the identity of the visual stimulus.

In total, 30 blocks were presented, distributed across the two sessions for all participants. Furthermore, per SOA there were 10 stimuli for every audio-visual combination for the five participants who saw the full range of SOAs, and 11 stimuli per SOA for the other three participants. Blocks lasted approximately 7 min each. Additionally, there were catch trials in which a visual or auditory unimodal stimulus (20 stimuli for each) was presented. During the auditory unimodal presentation randomly one of the still visual faces, which were also used during the fade in of the moving faces, was presented. During the visual unimodal presentation white noise was presented at the same intensity as the audio-visual trials and participants had to indicate the identity of the visual stimulus. This ensured that participants were actually looking at the screen.

Participants were seated approximately 57 cm from the screen and were instructed to look at the fixation cross at all times if presented. Presentation software (Neurobehavioral Systems, Inc., Albany, NY, USA) was used for stimulus presentation. Visual stimuli were presented on a gray background (RGB: 100, 100, 100). After each block participants were encouraged to take a break and it was ensured that participants never engaged continuously in the task for more than half an hour.

### Data analysis

With regard to the unimodal stimuli, we aimed to replicate previous findings stating that voicing is discriminated better in the auditory modality, whereas POA is discriminated better in the visual modality (Wiener and Miller, 1946; McGurk and MacDonald, 1976; Summerfield, 1987). For the analysis concerning voicing, the percentage of voiced responses was calculated per voicing category. Thereafter, we averaged the response proportions and performed an arcsine-square-root transformation to overcome non-normality caused by the restricted range of the proportion data (however in the figures proportions are kept for illustration purposes, since they are more intuitive). The calculated transformed response proportions per category were used as dependent variables in two repeated measurements ANOVAs, for the visual as well as for the auditory modality. For the visual unimodal analyses, the data from the unimodal visual experiment was used (although the data from the visual catch trials in the AV experiment gave comparable results), whereas for the auditory analyses the catch trials in the audio-visual experiment were analyzed. To investigate whether participants could identify the voicing of the stimulus the factors Voicing of the stimulus (voiced vs. unvoiced stimuli) and Voicing of the response were used. A similar analysis was performed to investigate whether POA could be identified in the auditory and visual modality. Here, the percentage of POA responses per POA category were calculated, arcsine-squared-root transformed, and the factors POA of the stimulus (bilabial, coronal, or dorsal) and POA of the response were used in two repeated measurements ANOVAs for the visual and auditory modality. For significant interactions simple effect analyses per stimulus category were performed. If not otherwise reported, all multiple comparisons were Bonferroni corrected and effects of repeated measures were corrected for sphericity issues by Greenhouse–Geisser correcting the degrees of freedom.

For the Audio-visual analyses, we first performed the same analyses as for the unimodal stimuli, collapsed over the SOAs, separately for visual /pa/ and /ga/. Thereafter, linear mixed models were used to investigate the SOA effects. This approach was chosen to accommodate for the missing data which arose because three participants were only presented with SOAs between VA 300 and AV 300 ms instead of VA 540–AV 540 ms. Per visual stimulus and per voicing level a mixed model was run with the factors Stimulus POA, Response (only responses that were on average per VC category above chance level were used for further analyses) and SOA. This factor was created by binning the differently used SOAs in nine bins with center points: VA 50, 125, 275, and 475, 0 and AV 50, 125, 275, and 475. These bins were chosen to include all the SOAs used. Additionally, a random intercept was added to account for the individual variations in the baseline.

**Figure 1:**
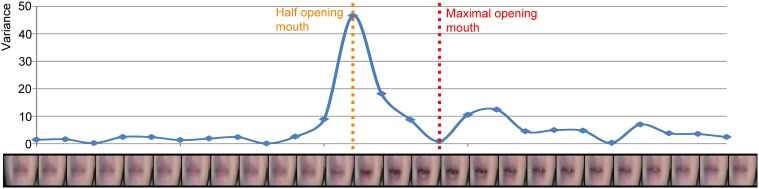
**Example of the envelope of the velocity of the mouth movement of visual /pa/**. Each dot represents the variance over all pixels of the mean RGB difference for two adjacent frames (the frame left and right of the dot). The orange dotted line represents the half opening of the mouth and the red dotted line represents the maximal opening of the mouth.

We hypothesized differential effects as a result of natural differences in onset asynchronies of mouth movements and congruent speech sounds, for example between dorsal (earlier movements) and coronal syllables (later movements). In order to investigate this hypothesis, we calculated the velocity of the mouth movements as follows. For each visual stimulus we zoomed in on the area around the mouth (see Figure 1). Then, the mean of the absolute differences of the three RGB values per pixel for adjacent frames was calculated. Thereafter, to quantify the movement from one frame to the other, the variance of the mean absolute RGB differences over the pixels was calculated and this was repeated for all the frames. This resulted in a velocity envelope of the mouth movement (i.e., comparable to the derivative of the mouth movement—it indicates *changes* in the movement) in which a clear opening and closing of the mouth becomes visible (see Figure 1). The result of this method is similar to the methods used by Chandrasekaran et al. (2009), such that the point of maximum velocity coincides with a half open mouth and the minimum velocity coincides with a fully open mouth. To quantify the onset differences between the auditory and visual signals, the time point of maximal amplitude of the auditory signal was subtracted from the time point of maximal velocity of the visual signal. These values were later used in a linear mixed model (see Results for details).

## Results

### Unimodal effects

**Figure 2:**
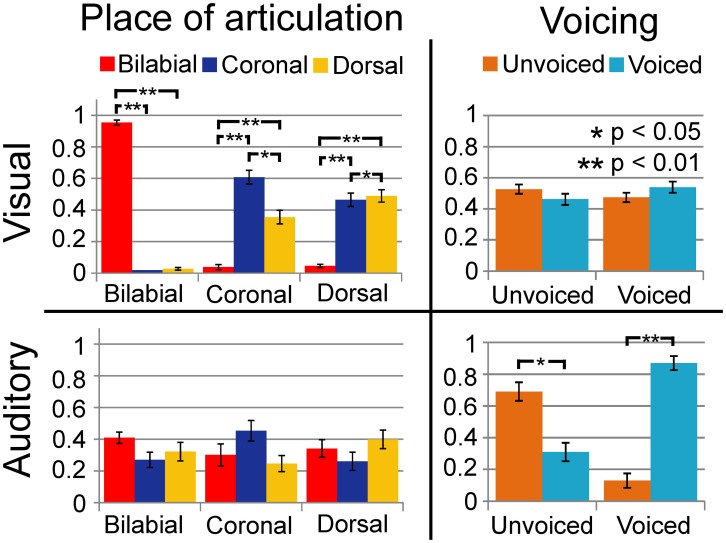
**Results of unimodal analyses for auditory and visual signals separately**. Horizontal axis represents the category of the stimulus and vertical axis represents the response proportions of the respective categories. Dashed lines indicate chance level performance. As shown, vision can dissociate place of articulation (POA) and audition can dissociate voicing (VC).

We replicated previous results showing that voicing is most optimally discriminated in auditory syllables and POA most optimally in visual syllables (see Figure 2; Tables 1 and 3). Table 1 indicates that the response POA interacts with the stimulus POA only for the visual stimuli, which means that for a stimulus with a specific POA the POA categories have different response proportions during the visual experiment. Simple effects show that especially bilabial stimuli were identified correctly during the visual experiment (as indicated by significantly higher bilabial than dorsal and coronal responses). Dorsal and coronal visual stimuli were more often confused with each other. However, for the unimodal auditory stimuli, the interaction between response and stimulus POA did not reach significance, indicating that participants were not able to dissociate the POA of the auditory stimuli. Table 3 (top rows) shows significant simple effects of the voicing of the response per stimulus level for the auditory, but not the visual modality. This means that in the auditory modality, voicing was primarily categorized correctly.

**Table 1:** **Results for the POA analyses of the unimodal stimuli**.

**(A)**		**POA interaction**	**Simple effects per stimulus level**								
			**Stimulus bilabial (B)**			**Stimulus coronal (C)**			**Stimulus dorsal (D)**		
			**B vs. C**	**B vs. D**	**C vs. D**	**B vs. C**	**B vs. D**	**C vs. D**	**B vs. C**	**B vs. D**	**C vs. D**
Auditory	F/t	2.34	–	–	–	–	–	–	–	–	–
	*P*	0.12									
Visual	*F/t*	178.4	23.2	26.8	−0.92	−9.89	−8.24	2.70	−9.6	−13.1	−0.16
	*P*	0.00^**^	0.00^**^	0.00^**^	1.00	0.00^**^	0.00^**^	0.09	0.00^**^	0.00^**^	1.00

The second column shows the interaction between stimulus and response place of articulation (POA interaction), and the other three columns show for stimuli with the different POAs the pairwise comparisons of the response proportions between the different POAs responses (B, bilabial; C, coronal; and D, dorsal). Auditory and visual rows indicate the results from the auditory only trials during the audio-visual experiment and the separate unimodal visual experiment, respectively. Results for post hoc analyses are only shown if ANOVA tests are significant.

** indicates p-values below 0.01.

### Multimodal effects collapsed over SOAs

**Figure 3:**
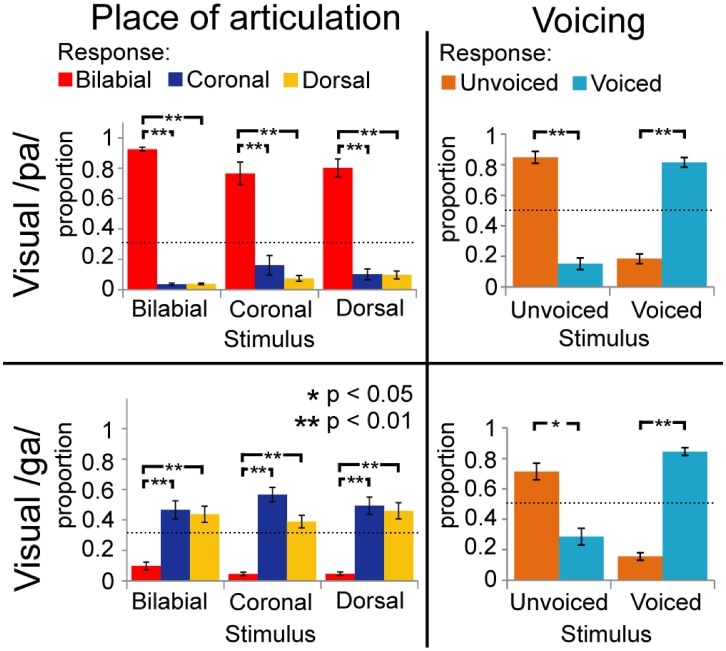
**Results of multimodal analyses for visual /pa/ and /ga/ separately collapsed over stimulus onset asynchronies (SOAs)**. Horizontal axis represents the category of the stimulus and vertical axis represents the response proportions of the respective categories. Dashed lines indicate chance level of responding. Voicing (VC) is dissociable, but place of articulation (POA) responses depended on the unimodal visual response in Figure 2.

During the audio-visual experiment, the voicing of the stimuli was identified correctly most of the time (as indicated by significant simple effects for the voicing analyses; see Figure 3; Table 3), and resembles the results from the unimodal auditory analyses. The results for the POA, when visual /pa/ was presented, resulted in high response proportions (more than 0.8) for bilabial stimuli (see Table 2), paralleling visual unimodal results. The POA response × stimulus interaction effect indicates that bilabial responses are specifically reported when the auditory stimuli is also bilabial, but in the simple effects the comparisons did not show significant differences (Table 2, row 3). Similarly, the response distributions for dorsal stimuli in the unimodal visual experiment and the visual /ga/ during the audio-visual experiment seem to resemble each other, that is, in the audio-visual experiment participants also confused the coronal and dorsal POA.

**Table 2:** **Results for the POA analyses of the multimodal stimuli**.

**(B)**		**POA interaction**	**Simple effect for congruent response**			**POA Response; main effect**	**Pairwise comparisons of response level**		
			**B vs. C**	**B vs. D**	**C vs. D**		**B vs. C**	**B vs. D**	**C vs. D**
AV, visual /pa/	*F/t*	6.30	2.41	2.23	−1.89	92.2	8.33	10.6	1.15
		0.02^*^	0.14	0.19	0 29	0.00^**^	0.00^**^	0.00^**^	1.00
AV, visual /ga/	*F/t*	3.43	–	–	–	39.78	−4.80	−7.94	0 03
	*p*	0.07				0.00^**^	0.01^**^	0 00^**^	1.00

The second column is similar as in Table 1. The third column shows the simple effect for the visual congruent response option (for visual /pa/ the bilabial response), comparing whether for specific stimuli the congruent visual POA option has a higher proportion. The fourth column shows the main effect of the response of the POA. The last column shows the pairwise comparisons whether overall, one POA response is given more often than another (B, bilabial; C, coronal; and D, dorsal). Results for post hoc analyses are only shown if ANOVA tests are significant.

* and ** indicate p-values below 0.05 and 0.01, respectively.

**Table 3:** **Results for voicing for both unimodal and multimodal stimuli**.

**(C)**		**Voicing interaction**	**Response simple effects per stimulus level: voiced vs. unvoiced**	
			**Stimulus voiced**	**Stimulus unvoiced**
Auditory	*F/t*	43.8	8.19	−2.83
	*p*	0.00^**^	0.00^**^	0.03^*^
Visual	*F/t*	18.5	1.66	−0.13
	*p*	0.00^*^	0 14	0.90
AV, visual /pa/	*F/t*	112	8.71	−6.82
	*p*	0.00^**^	0.00^**^	0.00^**^
AV, visual /ga/	*F/t*	87.2	11.42	−3.94
	*p*	0 00^**^	0.00^**^	0.01^**^

The second column is the interaction of stimulus voicing with response voicing (voicing interaction). The third and fourth columns are the simple effect analyses of the voicing of the response per stimulus level. Results for post hoc analyses are only shown if ANOVA tests are significant.

* and ** indicate p-values below 0.05 and 0.01, respectively.

**Figure 4:**
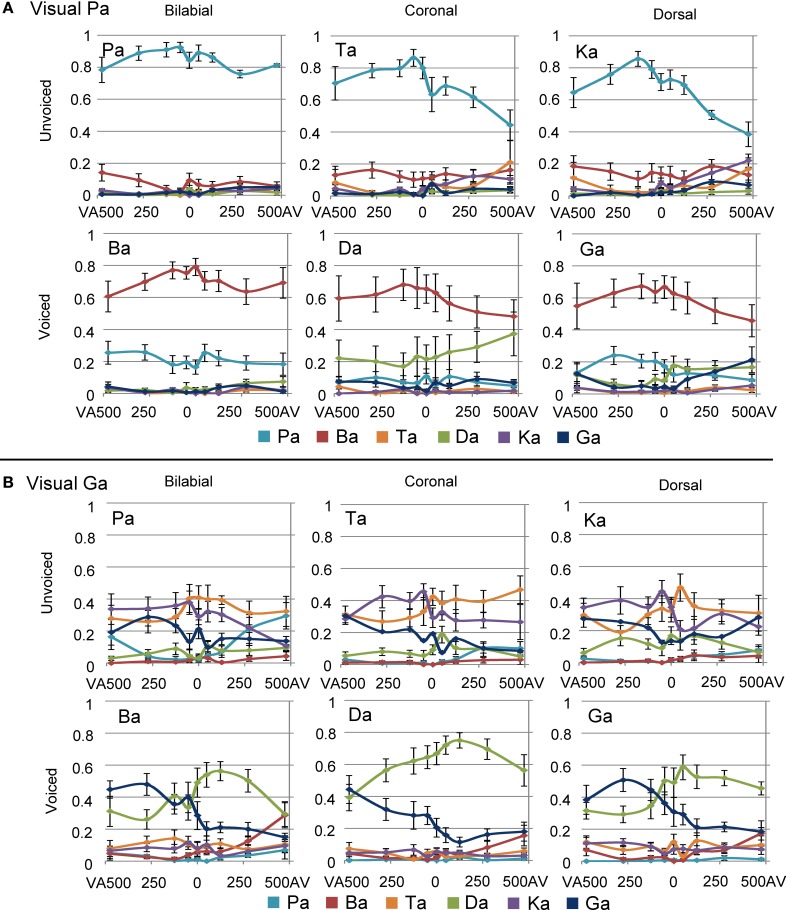
**Overall results of the multimodal experiment for visual /pa/ (A) and visual /ga/ (B), combined with the six auditory stimuli and all stimulus onset asynchronies (SOAs)**. Negative SOAs indicate that the visual stimulus was shifted to an earlier point in time compared to the auditory stimulus.

The latter analysis shows that adding a visual stimulus changes the auditory percept for the different POA categories, such that with incongruent audio-visual POA, the correct POA response choice (i.e., the POA of the auditory stimulus) is nearly absent in the chosen responses. For example, although a dorsal auditory stimulus is presented (e.g., /ka/), if concurrently visual /pa/ is presented, the response options with dorsal POAs are only chosen approximately 10% of the times (see Figures 3 and 4). Therefore, we decided that, for the analyses including the temporal factors, we would only use the response options that were given more than chance level per stimulus voicing and POA (POA: 0.33, voicing: 0.5). Mainly, because we were interested in the temporal pattern of the identification and a very low response rate could result in floor effects, biasing the statistical analyses. Thus for visual /pa/, auditory unvoiced we only used response /pa/ (see Figure 3; stimulus unvoiced and POA bilabial) and for visual /pa/, auditory-voiced we only used response /ba/ (stimulus voiced and POA bilabial). For visual /ga/, auditory-unvoiced response options /ta/ and /ka/ were used (stimulus unvoiced and POA coronal and dorsal respectively) and for visual /ga/, auditory-voiced response options /da/ and /ga/ were used (stimulus voiced and POA coronal and dorsal respectively).

### Temporal effects during visual /pa/

**Figure 5:**
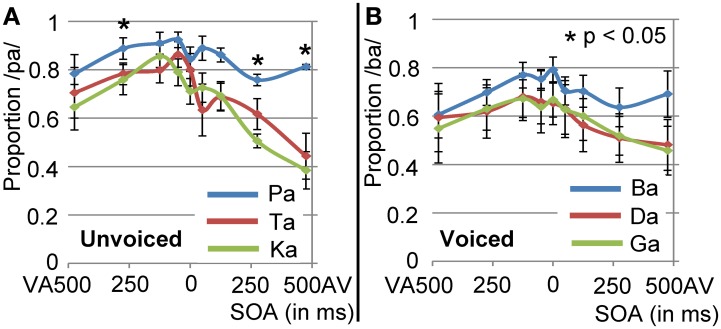
**Results for visual /pa/ presentation for unvoiced stimuli (A) and voiced (B) stimuli**. Only response proportions are shown for the response options that were given above chance level. These response options were /pa/ and /ba/, for unvoiced and voiced stimuli respectively.

Overall effects of SOA difference are shown in Figure 4. The mixed model analyses for visual /pa/, auditory unvoiced showed an main effect for POA and SOA [Figure 5A; *F*(2, 180) = 34.04, *p* < 0.001 and *F*(8, 180) = 10.88, *p* < 0.001, respectively]. Bilabial responses were reported significantly more than coronal and dorsal responses [*t*(180) = 7.60, *p* < 0.004 and *t*(180) = 6.59, *p* < 0.001, respectively]. The main effect of SOA indicated that compared to an SOA of zero, for AV 475 and AV 275 lower /pa/ response proportion were given [*t*(180) = −4.60, *p* < 0.001 and *t*(180) = −4.583, *p* < 0.001, respectively]. Thus, the proportion /pa/ responses were the least for incongruent bilabial presentation, and when auditory stimuli were leading more than 125 ms. Visual /pa/, auditory-voiced stimuli resulted in similar results: an main effect for POA and SOA [Figure 5B; *F*(2, 180) = 13.59, *p* < 0.001 and *F*(8, 180) = 4.83, *p* < 0.001, respectively]. Bilabial response proportions were higher than coronal and dorsal response proportions [*t*(180) = −4.49, *p* < 0.001 and *t*(180) = −4.54, *p* < 0.001, respectively]. Here, for a smaller window /ba/ responses were given compared to visual /pa/–unvoiced /pa/ responses, that is, the SOAs of AV 475, AV 275, and VA 475 were significantly different from an SOA of zero [AV 475: *t*(180) = −4.027, *p* < 0.001; AV 275: *t*(180) = −3.639, *p* = 0.003; and VA 475: *t*(180) = −3.584, *p* = 0.004].

### Temporal effects during visual /ga/

**Figure 6:**
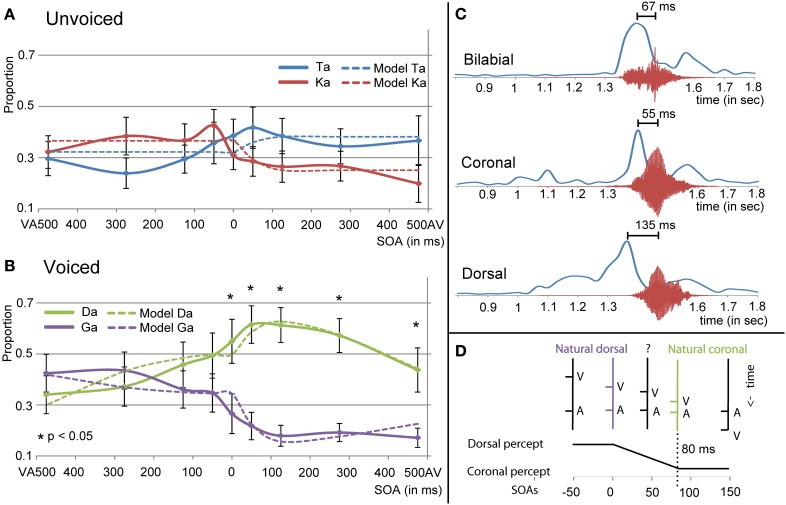
**Results for visual /ga/ presentation for unvoiced (A) and voiced (B) auditory stimuli. (C)** Shows the onset differences in visual velocity and auditory amplitude for the different place of articulations (POAs). **(D)** Shows the predictor for the mixed model analyses using the natural dorsal and coronal onset asynchronies. The fit of the model together with the other significant predictors in the mixed model analyses is represented in **(A,B)** as dashed lines.

The multilevel analyses for the visual /ga/ unvoiced showed an interaction effect between response and SOA [*F*(8, 371) = 4.540, *p* < 0.001]. Results from the simple effects analyses in which the /ta/ and /ka/ responses per SOA level were compared indicated that for SOA VA 275 /ka/ was indicated more and for SOA AV 50, 125, and 475 /ta/ was indicated more [uncorrected values: −275 = −2.813, *p* = 0.008; 50: *t*(24) = 2.088, *p* = 0.041; 125: *t*(24) = 2.394, *p* = 0.022; 475: *t*(24) = 2.650, *p* = 0.014], but these effects did not survive correction for multiple comparisons. The interaction effect however, seems to be caused by more answered /ka/ with negative SOAs, and more answered /ta/ with positive SOAs (see Figure 6A).

For the visual /ga/, auditory-voiced the multilevel analyses also showed an interaction of response and SOA [see Figure 6B; *F*(8, 367) = 11.996, *p* < 0.001]. Additionally, it showed an interaction between stimulus POA and response [*F*(8, 367) = 26.480, *p* < 0.001]. One explanation for this last effect could be that our [da] stimulus was better identifiable unimodally than the other auditory stimuli (see Figure 4), such that for stimulus POA coronal a higher proportion /da/ responses were given (since this was the right answer). This was similar during visual /pa/, auditory [da], which also showed a higher proportion /da/ compared to the correct responses during other incongruent combinations (Figure 4A). For the response × SOA interaction we performed simple effects analyses per SOA level. For all AV SOAs and SOA 0 /da/ was reported significantly more than /ga/ [475: *t*(24) = 4.667, *p* < 0.001; 275: *t*(24) = 7.624, *p* < 0.001; 125: *t*(24) = 9.089, *p* < 0.001; 50: *t*(24) = 6.615, *p* < 0.0001; 0: *t*(24) = 3.922, *p* = 0.004].

### “Crossing” identification for visual /ga/

Around the zero point, we observed a quick incline or decline in the response choice of participants for visual /ga/ (see Figures 4B and 6), such that participants chose with positive SOAs more often coronal responses (/da/ or /ta/) and with negative SOAs more often dorsal responses (/ga/ or /ka/). The decline seems to be less strong for visual /ga/, auditory [da]. This is probably related to the better unimodal auditory identification of auditory [da]. However, also here the incline for /ga/ responses and decline for /da/ responses around zero is observable. The “crossing” could relate to inherent differences in onsets between visual and auditory signals for coronal and dorsal stimuli. Indeed, a 2 × 3 ANOVA with factors POA and VC comparing onset differences between the maximal amplitude for visual velocity and auditory signal showed an effect of POAs [see Figure 6C; *F*(1, 12) = 8.600, *p* = 0.005]. Pairwise comparisons showed that dorsal stimuli had significantly bigger AV onset differences than coronal or bilabial stimuli [dorsal-coronal: *t*(5) = 2.757, *p* = 0.012; dorsal-bilabial: *t*(5) = 1.941, *p* = 0.033; bilabial-coronal: *t*(5) = 0.466, *p* = 1.000]. In our stimulus set we did not find a significant difference between voiced and unvoiced stimuli [*F*(1, 12) = 0.800, *p* = 0.389], so we collapsed this for further analyses and figures.

To model whether these inherent differences in onset asyn-chronies could explain the observed crossing, a new mixed model analysis was conducted. Therefore, we changed the factor SOA into a quantitative factor as described in Figure 6D. The logic of the model is as follows: since both unimodal stimuli alone cannot conclusively define the identity of the stimulus (auditory unimodal can differentiate voicing, but visual unimodal can only exclude bilabial), two options are left. Our perceptual system might resolve this issue by using another cue, namely time differences between audio-visual syllable pairs. In our stimulus set, a SOA of zero is equal to the onset asynchronies of dorsal stimuli, because we aligned the stimuli based on the maximal amplitude of auditory [ga] (see Figures 6C,D). The difference between dorsal and coronal onsets is on average 80 ms (average audio-visual asynchrony for dorsal is 135 ms and for coronal 55 ms). Therefore, the SOA for coronal stimuli in our stimulus set would be around +80 ms. With SOAs bigger than 80 ms the onset asynchronies match closer to coronal than to dorsal asynchronies. The opposite is true for audio-visual pairs with a long (experimental) visual lead: the onset asynchronies are close to dorsal asynchronies. In between these natural lags there is an ambiguity with regard to the identity of the stimulus. This factor therefore specifically tests our hypothesis that dependent on the audio-visual onset difference, participants would be biased in choosing the dorsal or coronal option, which provides new insight in the mechanism of how the percept is formed in case of ambiguous inputs. Additionally, we added a second order polynomial to the analyses to account for the downslope at the extremes.

The results of this mixed model showed an interaction between response and the created factor in both the unvoiced and voiced analyses [Figure 6B; *F*(1, 385) = 22.446, *p* < 0.001 and *F*(1, 379) = 58.166, *p* < 0.001, respectively], indicating that indeed modeling the natural lag in audio-visual syllables explains the difference in the response choice for the different SOA. In both voicing levels dorsal responses had positive and coronal responses negative values for the parameter estimate (Unvoiced: parameter estimate −0.1410 and 0.0689 for /ta/ and /ka/ respectively and Voiced: parameter estimate −0.2212 and 0.1674 for /da/ and /ga/ respectively), verifying the hypothesized pattern of the effect in which negative SOAs should result in a dorsal percept. As in the previous analyses, POA showed an interaction with response for the visual /ga/ stimulus [*F*(2, 379) = 26.731, *p* < 0.001]. The second order factor was only of significance in the analyses with the voiced stimuli and showed an interaction with response [*F*(1, 379) = 22.279, *p* < 0.001], such that the parameter estimate was more negative for the /ga/ response.

## Discussion

The current study investigated the influence of content and temporal cues on the identification of audio-visual syllables. We hypothesized that visual input influences the percept only within a constrained temporal window. Furthermore, we predicted that the more reliable unimodal content cues determine the percept more strongly. Finally, we hypothesized that information about natural audio-visual onset differences can be used to identify syllables. We revealed that during audio-visual speech perception visual input determines the POA and auditory input determines the voicing. Moreover, we confirmed the prediction of a wide window in which visual information influences auditory perception that was wider for congruent stimulus pairs. Interestingly, within this window, the syllable percept was not consistent, but differed depending on the specific SOA. This was particularly pronounced when the POA could not be reliably identified (i.e., between dorsal and coronal stimuli). We explained this temporal response profile using information about natural onset differences between the auditory and visual speech signals, which are indeed different for the dorsal and coronal syllables.

### Multiple unimodal cues for audio-visual speech identification

Our data suggest that participants used the visual signal to identify the POA and the auditory signal to identify voicing during audio-visual presentation. We suggest that it is the reliability of the cue for the specific features of the syllable that determined the percept, since it has been shown before that the reliability of a cue can determine the percept (Massaro, 1997; Andersen et al., 2004). This is also in line with our replication of the results that unimodally, visual stimuli are best dissociable by using POA and auditory stimuli are best dissociable by using voicing (Wiener and Miller, 1946; Summerfield, 1987; van Wassenhove et al., 2005). It appears that irrespective of the task, which was to identify the auditory stimulus, visual input influences perception. Therefore, it seems that audio-visual speech is automatically integrated, since participants were not able to perform the task using only auditory cues as instructed. Integration in our study is shown by different identification responses for auditory and audio-visual presentation of the same spoken syllables. This perceptual effect is similar to the McGurk effect, in which identification of an auditory syllable is involuntarily influenced by an incongruent visual input (Soto-Faraco et al., 2004; Gentilucci and Cattaneo, 2005). This indicates that during audio-visual speech perception, an integrated percept is created that uses the information of the visual as well as the auditory domain. In the current setting, since the auditory signal is non-optimal, this leads to a considerable bias in favor of the visual POA, for which the visual input is most reliable and thus dominant. In the McGurk effect, both signals are equally salient, resulting in a fused percept. So, when a unimodal signal is dominant during audio-visual integration, this predisposes perception.

### Content predictions in audio-visual speech

In the current study we manipulated the predictability of the visual signal by using one visual syllable in which the POA can reliably be determined (/pa/) and another syllable in which the POA estimate is less reliable (/ga/). Previous research has shown that the information present in the visual signal is used to determine our percept, for example, van Wassenhove et al. (2005) showed facilitation of congruent speech dependent the amount of content information in the visual stimuli. Consistent with our results, van Wassenhove and colleagues showed that, /pa/ stimuli which convey more content information about POA, influenced electro-encephalographic recordings more than a less informative syllable /ka/. In their study, an analyses-by-synthesis framework was proposed in which the auditory signal is evaluated, based on the predictive strength the visual signal has for the content of the auditory signal. This predictive strength should determine whether there is a McGurk effect (van Wassenhove et al., 2005) and should also correlate with prediction error when an incongruent auditory stimulus is presented (Arnal et al., 2011). In a study using congruent audio-visual speech with auditory speech in white noise, Pandey et al. (1986) showed that more proficient lip readers can still detect the auditory signal at higher noise levels, indicating that the predictive strength or the amount of information conveyed by the visual signal, influences the amount of benefit during auditory perception. Here, we also show that more predictable visual bilabial stimuli bias the percept more strongly, because visual /pa/ shaped the percept more profoundly than visual /ga/. This is in line with results from Vatakis et al. (2012) who found that the point of perceived synchrony needed more visual lead for stimuli pronounced more in the back of the mouth compared to bilabial stimuli. They argue that for more salient visual stimuli (i.e., bilabial stimuli) a smaller visual lead is required to reach synchrony perception. In our study, this is reflected in the amount of bias of the visual signal for the POA response choice. Since the auditory signal had a low signal to noise ratio, the visual signal biases the percept of POA completely, such that unimodal and audio-visual POA response proportions were the same.

### Interplay between two distinct temporal cues in audio-visual speech perception

It is well-known that temporal cues are informative for audiovisual speech identification (Munhall and Vatikiotis-Bateson, 2004; Zion Golumbic et al., 2012). Firstly, auditory and visual speech seems to temporally co-vary (Campbell, 2008). Especially in theta frequencies around 2–7 Hz, lip movement and the auditory envelope seem to correlate (Müller and MacLeod, 1982; Chandrasekaran et al., 2009; Luo et al., 2010). This feature has been considered a main source of binding and of the parsing of information (Poeppel, 2003; Campbell, 2008; Ghazanfar et al., 2013) and removing this frequency reduces auditory intelligibility (Vitkovitch and Barber, 1994; Ghitza, 2012). Secondly, visual signals generally precede auditory signals, providing temporal predictability of the arrival of the auditory signal (Schroeder et al., 2008). Finally, audio-visual speech perception has generally been shown to have a broad integration window (Dixon and Spitz, 1980; Grant and Greenberg, 2001), which has led to the conclusion that audio-visual speech perception has loose temporal associations (Munhall and Vatikiotis-Bateson, 2004). Our results also indicate that visual input influences the auditory percept for a wide range of SOAs. For example, we show that with auditory [ba] and visual /ga/, the visual signal influences the percept for a time window in which the visual signal is shifted 500 ms earlier in time, relative to the auditory signal, up to when the visual signal was shifted 300 ms later in time, relative to the auditory signal (SOAs ranging from VA 500 up to AV 300 ms). Only at the most positive SOA (AV 500) is visual information not used and the correct answer [ba] is present in the given responses.

**Figure 7:**
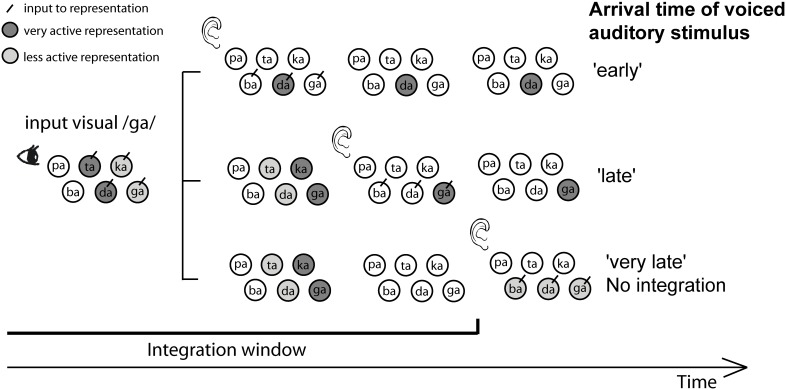
**Proposed mechanism explaining the interplay between place of articulation (POA), voicing, temporal integration, and temporal onset cues**. The figure shows what happens during the presentation of visual /ga/ and an ambiguous voiced stimulus. For the visual syllable /ga/, POA cues present in the visual signal activate (indicated by darker circles) coronal (/ta/ and /da/) and dorsal (/ka/ and /ga/) representations in a time-dependent manner: the activation decays over time (indicating the TWI), and depending on the natural audio-visual onset differences, maximal activation occurs at different time points for the two POAs (later for dorsal than for coronal). Therefore, the time when auditory information activates representations of syllables (represented along the vertical axis) is important for winning the decision making process. When auditory syllables arrive early, and therefore resemble more closely natural audio-visual onset differences for /da/, /da/ is more active than /ga/, and has the highest chance to win the decision making process. In this example, the visual cues can not distinguish between the different coronal and dorsal possibilities, and the auditory cues cannot distinguish the POA at all, so the arrival of the auditory information (early vs. late) facilitates this decision; early onset will activate the coronal /da/ and late onset will activate the dorsal /ga/ syllable.

Although we find integration during a wide window, the results do not support a very loose temporal association, since we also found evidence for the use of natural temporal audio-visual onset differences in identifying the syllable. However, this information was only used when unimodal cues did not provide enough information. Therefore, we propose the following mechanism for the interplay of articulatory cues (POA and voicing), temporal integration cues, and temporal onset cues (see Figure 7): first, the visual and auditory components of a syllable activate syllable representations based on their “preferred” cue and reliability. However, these activations have some decay, such that at some point in time after the visual stimulus was presented, visual information does not influence the percept anymore (the TWI). Within this window more reliable cues will cause more activation of specific representations (i.e., visual cues will activate representations of syllables with corresponding POAs and auditory cues will activate representations of syllables with corresponding voicing). In a winner-takes-all framework, which is the case in an identification task, only one representation can win and that will be the representation with the strongest input. However, in addition to the visual and auditory articulatory cues, the activation of syllable representation is also based on the encoded natural onset differences. That is, for dorsal stimuli (e.g., /ga/), maximal activation will occur later than for coronal stimuli (e.g., /da/). When an ambiguous auditory stimulus arrives, it will activate multiple representations (the three voiced representations in the figure). The representation that is most active at that point in time, depending on the audio-visual onset difference, will win the competition. In the figure, visual /ga/ input cannot dissociate the coronal (/da/ and /ta/) from the dorsal (/ga/ and /ka/) POA, and auditory information cannot dissociate the POA at all. Therefore, if the auditory stimulus arrives early (resembling natural coronal audio-visual onset differences), the most active representation will win the competition, in this example /da/. For later presentation, /ga/ will be more activated, and when the decay is completed there is no bias from the visual cue (since no representations are active), and one of the three voiced stimuli has to be chosen. This way, audio-visual onset differences only influence identification when ambiguous auditory stimuli are presented within the TWI, and only if the visual POA cues are not decisive.

### Temporal window of integration is influenced by audio-visual congruency

The TWI is generally measured by evaluating whether participants can indicate if audio-visual events are presented simultaneously or not (Vroomen and Keetels, 2010), assuming that when participants can reliably dissociate the two, the audio-visual event is perceived as two separate events and not bound together. However, little research has been done to assess whether audio-visual SOA differences also influence unimodal perception, which was one of the aims of the current study. Applying the same logic as that used for simultaneity judgments, events that are bound should influence unimodal perception more than when they are perceived separately. We here show that especially during congruent audio-visual voicing (visual /pa/, auditory unvoiced), the response proportions of /pa/ are higher (Figure 5). Also, visual influence seems to have a wider TWI for the congruent pairing of visual /pa/ with auditory /pa/, as the visually determined /pa/ response proportion appears higher for a wider temporal window (although the statistical test did not show this). One explanation for these congruency effects is the “unity assumption” stating that when two stimuli naturally belong together they are bound more strongly and therefore are more difficult to dissociate over a wider temporal window (Welch and Warren, 1980). However, it could be that with extreme SOAs, visual information is not used and participants rely only on the auditory signal, that is, in the case of congruent audio-visual /pa/ pairing they would also report /pa/ with auditory presentation only. Nonetheless, the unimodal auditory experiment showed that the POA for unvoiced stimuli could not be dissociated, neither could it for /pa/. Thus, the use of auditory information alone should not result in a higher proportion of /pa/ responses. For the incongruent pairs, identification with the most positive SOA seems similar to uni-modal unvoiced auditory perception, hence participants did not seem to use visual information, indicating that for this SOA integration did not take place. Similar results have been found by Vatakis and Spence (2007), who showed that judging simultaneity is more difficult when the gender of the speaker is congruent with the speech sound. Although there are also conflicting results, for speech the unity assumption seems plausible (Vroomen and Keetels, 2010).

One difference between simultaneity judgments and stimulus identification across SOAs seems to be that the point of maximal integration is more biased toward visual leading when explicitly asking about identity (Zampini et al., 2003; van Wassenhove et al., 2007). Therefore, varying SOAs and measuring unimodal perception might provide a different approach to measure whether integration occurs over a broader range of SOAs. This approach does not investigate whether two stimuli are perceived as simultaneously, but serves the goal to investigate the temporal patterns in which a unimodal stimulus influences the perception of another unimodal stimulus, for example the content of a stimulus. This judgment might be more natural, since in daily life, identifying stimuli is a more common act than explicitly judging their coincidence.

### Possible neuronal mechanisms

Based on previous literature, the brain area most consistently involved in audio-visual integration is the posterior superior temporal sulcus (Calvert and Lewis, 2004). It has been found active during visual and audio-visual speech perception (Calvert et al., 1997; Callan et al., 2004), seems to be sensitive for congruent vs. incongruent speech signals (Calvert et al., 2000; van Atteveldt et al., 2004, 2010), and responds to audio-visual onset differences (van Atteveldt et al., 2007; Chandrasekaran and Ghazanfar, 2009). In the temporal domain it seems that different temporal features (co-variations between mouth velocity and speech envelope and visual-auditory speech onset differences) have to be combined to shape our percept. Chandrasekaran and Ghazanfar (2009) showed that different frequency bands are differently sensitive for faces and voices in superior temporal cortex. Although theta oscillations have been shown to be influenced by input from other senses (Lakatos et al., 2007; Kayser et al., 2008), they have not been shown to have specific effects dependent on the voice-face onset differences and might therefore mostly be used to parse the auditory signals, enhance auditory processing, and might even relate to the audio-visual TWI (Poeppel, 2003; Schroeder et al., 2008). However, higher frequency oscillations have been shown to vary dependent on voice-face onset differences, and might be involved in encoding the identity of a syllable, thus explaining the current results. This is consistent with the notion that the auditory speech system depends on theta as well as gamma frequencies (Poeppel, 2003), and this latter time-scale might also be important in coding differences in natural audio-visual onset differences, and its influence on perception. These temporal constraints however would have to be investigated, for example by using combined behavioral and electrophysiological measures, or using transcranial magnetic stimulation at varying time points.

## Conclusion

Our findings show that within the integration window, visual information biases the auditory percept, specifically regarding the features in which the auditory signal is ambiguous (i.e., POA). Additionally, these findings indicate that natural temporal onset differences between auditory and visual input have a noteworthy influence on auditory perception. Although visual input has an influence over a wide temporal window during our experiment, we show that this initial binding of information does not conclusively determine our percept. Instead, it serves as a prerequisite for other interaction processes to occur that eventually form our perceptual decision. The final percept is determined by the interplay between unimodal auditory and visual cues, along with natural audio-visual onset differences across syllables. These results shed light on the compositional nature of audio-visual speech, in which visual, auditory, and temporal onset cues are used to create a percept. This interplay of cues needs to be studied further to unravel the building blocks and neuronal basis of audio-visual speech perception.

### Conflict of interest statement

The authors declare that the research was conducted in the absence of any commercial or financial relationships that could be construed as a potential conflict of interest.
